# Systematic review and meta-analysis of the prognostic impact of cancer among patients with acute coronary syndrome and/or percutaneous coronary intervention

**DOI:** 10.1186/s12872-020-01352-0

**Published:** 2020-01-30

**Authors:** Vincent Roule, Laurine Verdier, Katrien Blanchart, Pierre Ardouin, Adrien Lemaitre, Mathieu Bignon, Rémi Sabatier, Joachim Alexandre, Farzin Beygui

**Affiliations:** 1 CHU de Caen Normandie, Service de Cardiologie, Caen University Hospital, Avenue Cote de Nacre, 14033 Caen, France; 2 Normandie Univ, UNICAEN, EA 4650 Signalisation, électrophysiologie et imagerie des lésions d’ischémie-reperfusion myocardique, 14000 Caen, France; 3 CHU de Caen Normandie, Service de Pharmacologie, 14000 Caen, France

**Keywords:** Cancer, Prognosis, Acute coronary syndrome, Percutaneous coronary intervention

## Abstract

**Background:**

Patients with cancer admitted for an acute coronary syndrome (ACS) and/or percutaneous coronary intervention (PCI) represent a growing and high-risk population. The influence of co-existing cancer on mortality remains unclear in such patients. We aimed to assess the impact of cancer on early and late, all-cause and cardiac mortality in the setting of ACS and/or PCI.

**Methods:**

We performed a systematic review and meta-analysis of studies comparing outcomes of patients with and without a history of cancer admitted for ACS and/or PCI.

**Results:**

Six studies including 294,528 ACS patients and three studies including 39,973 PCI patients were selected for our meta-analysis. Patients with cancer had increased rates of in-hospital all-cause death (RR 1.74 [1.22; 2.47]), cardiac death (RR 2.44 [1.73; 3.44]) and bleeding (RR 1.64 [1.35; 1.98]) as well as one-year all-cause death (RR 2.62 [1.2; 5.73]) and cardiac death (RR 1.89 [1.25; 2.86]) in ACS studies. Rates of long term all-cause (RR 1.96 [1.52; 2.53]) but not cardiac death were higher in cancer patients admitted for PCI.

**Conclusion:**

Cancer patients represent a high-risk population both in the acute phase and at long-term after an ACS or PCI. The magnitude of the risk of mortality should however be tempered by the heterogeneity among studies. Early and long term optimal management of such patients should be promoted in clinical practice.

## Introduction

Coronary artery disease and cancer are the leading causes of mortality worldwide [1]. Cancer-related mortality has declined over the past decades due to earlier detection and advances in treatment [2, 3]. Consequently, patients with a history of cancer represent a growing population in general.

Patients with coronary artery disease and cancer often share common risk factors such as advanced age, sedentary lifestyle and smoking [4]. Anticancer therapies such as radiotherapy [5, 6] or drugs [7] are associated with an increased risk of coronary disease including myocardial infarction. Therefore, rates of history of cancer in patients admitted for an acute coronary syndrome (ACS) or elective percutaneous coronary intervention (PCI) are increasing in clinical practice but the data about the impact of a history of cancer on all-cause and more specifically cardiac mortality remain limited.

The aim of this study was to assess the in-hospital and long-term mortality among patients with and without a history of cancer using a systematic review and meta-analytic approach of published data in the setting of ACS and/or PCI.

## Methods

### Search strategy and studies’ selection

We followed the Preferred Reporting Items for Systematic Reviews and Meta-Analysis (PRISMA) guidelines for the systematic review and meta-analysis. We conducted a systematic literature review by formal searches of the electronic databases MEDLINE (source PubMed) and the Cochrane Controlled Clinical Trials Register Database through September 2018. Relevant trials were identified by a combination of medical subject headings including the following terms: acute coronary syndrome, myocardial infarction, acute myocardial infarction, non-ST elevation myocardial infarction, ST elevation myocardial infarction, cancer, neoplasm, mortality, outcomes and prognosis. References from reviews and selected articles were also reviewed for potential relevant citations. Studies were selected by 2 independent reviewers (VR and LV).

We restricted our analysis to the trials that met all of the following inclusion criteria: (1) comparison between patients with history of cancer, active or not (cancer group) and non-cancer patients; (2) patients admitted for an ACS and/or PCI; (3) available data on mortality. We excluded studies with no comparison between cancer and non-cancer patients.

### Outcomes

The primary outcomes assessed by the study were all-cause and cardiac in-hospital mortality. One study [8] reported 30-day mortality which was considered as early hence gathered with in-hospital mortality in the analysis. In-hospital bleeding, as defined in each study, was also included in the analysis. Long-term mortality was based on the longest follow-up available for each study.

### Assessment of risk of bias

We used the Newcastle-Ottawa Scale for assessment of risk of bias. This scale assesses risk of bias in the following 3 domains: selection of the study groups, comparability of groups, and ascertainment of exposure. Studies with scores of less than 4 were considered to have a high risk of bias, those with scores of 4 to 6 an intermediate risk of bias, and those with scores of 7 or more a low risk of bias.

### Statistical analysis

The total numbers of patients experiencing or not the outcomes of interest in each arm extracted directly from the publications were used for the analyses. Results are presented as relative risks (RR) with 95% confidence intervals (CI). Outcomes from individual studies were combined using the Mantel–Haenzel fixed and random-effect models. Heterogeneity across studies was studied by the Cochran’s Q statistic with a *p* value set at 0.1. The I^2^ was also taken into account regardless of the p value. An I^2^ of ≥50% was the pre-specified threshold considered too high to provide consistent analysis. The random-effect model was considered for the primary analysis. A fixed effect-model was also reported in figures, considered as a sensitivity analysis only. Tests were two-tailed and a *p*-value < 0.05 was considered statistically significant. Funnel plots and Egger’s regression test were used to assess publication bias. R software version 3.5.2 (2018-12-20) for MacOS (R Foundation for Statistical Computing) with Meta package was used for the analysis.

## Results

**Fig. 1:**
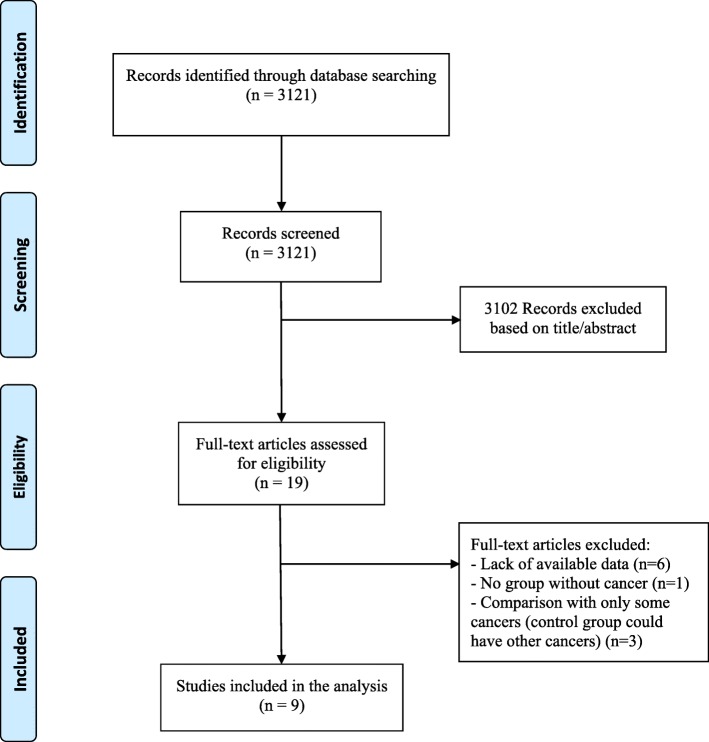
Flow diagram of meta-analysis studies’ selection

A total of 9 studies were selected for the meta-analysis: 6 studies [8–13] in the setting of ACS and 3 studies [14–16] in the setting of PCI (elective or for ACS), including 294,528 and 39,973 patients respectively. The review process is depicted in Fig. 1. Most studies were registries [9, 11–14, 16], one was obtained from databases [8] and the two remaining from retrospective cohorts [10, 15]. Two studies reported only pair-matched comparison results [11, 15] and two others used propensity scores [8, 14]. In one study [13] results were available in matched and unmatched groups: our principal meta-analysis was performed using the unmatched group as more endpoints were available and most included studies were not matched (the sensitivity analysis with the matched group of this study is reported in the Additional file 1). For one PCI study [16], the results of the subgroup of patients who underwent PCI for ACS were available and included in the ACS meta-analysis (named Nakastuma-AMI).

The major characteristics of the patients of each study are detailed in Table 1 for ACS studies and Table 2 for PCI studies. Overall, cancer patients represented 8.1% (5.6 to 23.4%) and 6.5% (3.3 to 9.1%) of all patients in the ACS and PCI studies respectively. Duration of long-term follow-up ranged between 5.3 and 11 years [8, 14–16].

**Table 1:** Demographic characteristics of patients with acute coronary syndrome in selected studies

	Velders et al.		Ianaccone et al.		Wang et al.		Kurisu et al.		Rohrmann et al.		Gong et al.	
Characteristics, n (%)	Cancer *n* = 208	No cancer *n* = 3215	Cancer *n* = 858	No cancer *n* = 13,773	Cancer *n* = 263	No cancer *n* = 2083	Cancer *n* = 18	No cancer *n* = 59	Cancer n = 1981	No cancer *n* = 1981	Cancer *n* = 22,907	No cancer *n* = 247,182
Age (years)	69.6 ± 11	62.8 ± 12.4	70.8 ± 10.3	62.8 ± 12.6	72.0 ± 10.7	63.3 ± 13.7	70.4 ± 8.1	68.8 ± 11.8	73.1 ± 11.6	73.1 ± 10.9	n/a	n/a
Men	141 (67.8)	2427 (75.5)	612 (71.3)	10,633 (77.2)	178 (67.7)	1480 (71.1)	13 (72.2)	44 (74.6)	1432 (72.3)	1443 (72.8)	13,996 (61.1)	153,747 (62.2)
Systemic hypertension	88 (42.7)	1135 (35.4)	558 (65)	7961 (57.8)	199 (75.7)	1429 (68.5)	11(61.1)	37 (62.7)	1309/1879 (69.7)	1313/1878 (69.9)	13,790 (60.2)	147,568 (59.7)
Diabetes mellitus	23 (11)	361 (11.2)	246 (28.7)	3237 (23.5)	44 (16.7)	381 (18.3)	7 (38.9)	25 (42.4)	439/1894 (23.2)	476/1890 (25.2)	6849 (29.9)	72,177 (29.2)
Hyperlipidemia	46 (22.4)	738 (23.1)	414 (48.2)	7327 (53.2)	186 (70.7)	1536 (73.7)	n/a	n/a	974/1704 (57.2)	1033/1737 (59.5)	8980 (39.2)	97,390 (39.4)
Active smoker	61 (31)	1487 (46.8)	n/a	n/a	169 (64.3)	1422 (68.3)	n/a	n/a	456/1728 (26.4)	494/1733 (28.5)	n/a	n/a
History of												
Myocardial infarction	35 (17)	335 (10.4)	132 (15.4)	1584 (11.5)	44 (16.7)	301 (14.5)	n/a	n/a	444/1981 (22.4)	404/1981 (20.4)	n/a	n/a
PCI	21 (10.2)	267 (8.3)	125 (14.6)	1708 (12.4)	55 (20.9)	330 (15.8)	n/a	n/a	n/a	n/a	916 (4)	9640 (3.9)
CABG	5 (2.4)	79 (2.5)	40 (4.7)	427 (3.1)	32 (12.2)	146 (7)	n/a	n/a	n/a	n/a	n/a	n/a
Stroke	24 (11.7)	191 (5.9)	71 (8.3)	744 (5.4)	39 (14.8)	161 (7.7)	n/a	n/a	164/1981 (8.3)	153/1981 (7.7)	1558 (6.8)	15,573 (6.3)
Anemia	53 (11.8)	152 (5.2)	n/a	n/a	n/a	n/a	9 (50)	4 (6.8)	312/807 (38.7)	192/777 (24.7)	4971 (21.7)	49,931 (20.2)
Clinical presentation												
STEMI	208 (100)	3215 (100)	440 (51.3)	8043 (58.4)	263 (100)	2083 (100)	n/a	n/a	1033/1981 (52.1)	987/1981 (49.8)	n/a	n/a
NSTEMI	0	0	279 (32.5)	3912 (28.4)	0	0	n/a	n/a	n/a	n/a	n/a	n/a
Unstable angina	0	0	139 (16.2)	1818 (13.2)	0	0	n/a	n/a	n/a	n/a	n/a	n/a
Number of diseased vessels												
1 vessel	87 (41.8)	1522 (47.4)	n/a	n/a	244 (92,8)	1959 (94)	9 (50)	35 (59.3)	n/a	n/a	n/a	n/a
2 vessels	71 (34.1)	1005 (31.3)	n/a	n/a	19 (7.2)	119 (5.7)	7 (38.9)	17 (28.8)	n/a	n/a	n/a	n/a
3 vessels	50 (24)	685 (21.3)	n/a	n/a	0	5 (0.2)	2 (11.1)	7 (11.9)	n/a	n/a	n/a	n/a
Procedure												
Stenting	192 (92.3)	3082 (95.9)	812 (94.6)	13,332 (96.8)	n/a	n/a	16 (88.9)	54 (91.5)	n/a	n/a	n/a	n/a
Drug-eluting stent	124 (60.8)	2263 (70.8)	304 (35.4)	5523 (40.1)	110 (41.8)	1138 (54.6)	1 (5.6)	7 (11.9)	n/a	n/a	n/a	n/a
Bare metal stent	67 (32.8)	812 (25.4)	508 (59.2)	7809 (56.7)	n/a	n/a	15 (83.3)	47 (79.7)	n/a	n/a	n/a	n/a
Treatments at discharge												
Beta-blockers	160 (84.2)	2812 (90.5)	640 (74.6)	11,198 (81.3)	n/a	n/a	2 (14.3)	27 (48.2)	1183/1960 (60.4)	1192/1957 (60.9)	7193 (31.4)	75,638 (30.6)
ACE/ARBs	139 (73.2)	2220 (71.5)	605 (70.6)	10,344 (75.1)	n/a	n/a	6 (42.9)	43 (76.8)	995/1961 (50.7)	1038/1962 (52.9)	8705 (38)	90,963 (36.8)
Statins	171 (90)	2897 (93.2)	780 (90.9)	12,878 (93.5)	n/a	n/a	8 (57.1)	41 (73.2)	1322/1958 (67.5)	1384/1965 (70.4)	5933 (25.9)	62,537 (25.3)
Aspirin	187 (98.4)	3097 (99.3)	850 (99.1)	13,690 (99.4)	n/a	n/a	13 (92.9)	56 (100)	1829/1973 (92.7)	1858/1975 (94.1)	n/a	n/a
P2Y12 inhibitors	186 (97.9)	3058 (98)	766 (89.3)	11,996 (87.1)	n/a	n/a	n/a	n/a	1380/1963 (70.3)	1465/1971 (74.3)	962 (4.2)	10,135 (4.1)

*ACE* Angiotensin-Converting Enzyme; *ARBs* Angiotensin Receptor Blockers; *CABG* Coronary Artery Bypass Grafting; *NSTEMI* Non ST-Elevation Myocardial Infarction; *PCI* Percutaneous Coronary Intervention; *STEMI* ST-Elevation Myocardial Infarction; *n/a* not available

**Table 2:** Demographic characteristics of patients admitted for percutaneous coronary intervention in selected studies

	Hess et al.		Nakatsuma et al.		Landes et al. (*)	
Characteristics, n (%)	Cancer *n* = 496	No cancer *n* = 14,512	Cancer *n* = 1109	No cancer n = 11,071	Cancer *n* = 969	No cancer n = 969
Age (years)	68 (61.8)	62 (53.7)	73.2 ± 8.5	67.8 ± 11.1	76.6 ± 10.1	76.9 ± 9.2
Men	354 (71.4)	9586 (66.1)	825 (74.4)	7976 (72)	700 (72.2)	700 (72.2)
Systemic hypertension	334 (67.3)	9478 (65.3)	904 (81.5)	9100 (82.2)	843 (87)	843 (87)
Diabetes mellitus	129 (26)	4013 (27.7)	440 (39.7)	4154 (37.5)	318 (45.7)	318 (45.7)
Hyperlipidemia	n/a	n/a	n/a	n/a		
Active smoker	238 (48)	7712 (53.1)	230 (20.7)	3648 (33)	230 (23.7)	204 (21.1)
History of						
Myocardial infarction	246 (49.6)	7414 (51.1)	119 (10.7)	1141 (10.3)	n/a	n/a
PCI	n/a	n/a	n/a	n/a	n/a	n/a
CABG	n/a	n/a	n/a	n/a	185 (19.1)	196 (20.2)
Stroke	66 (13.3)	1196 (8.2)	142 (12.8)	1149 (10.4)	87 (9)	63 (6.5)
Anemia	n/a	n/a	228 (20.6)	1165 (10.5)	n/a	n/a
Presentation						
ACS	329 (66.6)	10,481 (72.4)	317 (28.6)	2992 (27)	592 (61.1)	527 (54.4)
Number of diseased vessels						
1 vessel	255 (55.8)	8839 (65.4)	n/a	n/a	163 (16.9)	175 (18.1)
2 vessels	131 (28.7)	3095 (22.9)	n/a	n/a	320 (33)	310 (32)
3 vessels	71 (15.5)	1574 (11.7)	n/a	n/a	485 (50.1)	369 (40.9)
Procedure						
Drug-eluting stent	164 (54.5)	4209 (62.1)	570 (51.4)	6218 (56.2)	392 (40.5)	465 (48)
Bare metal stent	n/a	n/a	n/a	n/a	529 (54.6)	464 (47.9)
Treatment at discharge						
Beta-blockers	447 (90.1)	13,077 (90.1)	294 (27)	3410 (31)	n/a	n/a
ACE/ARBs	418 (84.3)	10,992 (75.7)	571 (51)	6573 (59)	n/a	n/a
Statins	n/a	n/a	487 (44)	5816 (53)	n/a	n/a
Aspirin	489 (98.6)	14,355 (98.9)	1092 (98)	10,936 (99)	n/a	n/a
P2Y12 Inhibitors	437 (88.1)	11,023 (76)	136 (13)	1018 (9.3)	n/a	n/a

*ACE* Angiotensin-Converting Enzyme; *ACS* Acute Coronary Syndrome; *ARBs* Angiotensin Receptor Blockers; *CABG* Coronary Artery Bypass Grafting; *PCI* Percutaneous Coronary Intervention

(*) data of the matched patients

**Fig. 2:**
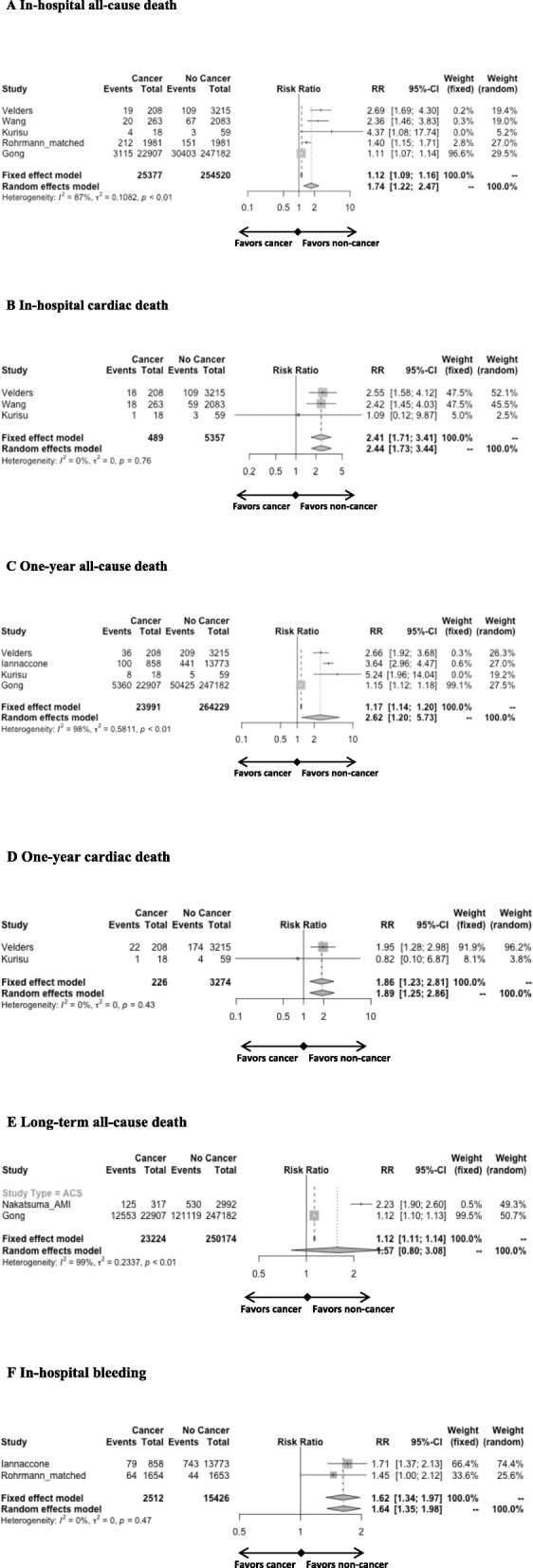
Forest plots of ACS studies comparing the impact of cancer versus no cancer on in hospital all-cause death (**A**), in-hospital cardiac death (**B**), one-year all-cause death (**C**), one-year cardiac death (**D**), long-term all-cause death (**E**) and in-hospital bleeding (**F**)

The analysis showed increased rates of in-hospital all-cause death (RR 1.74 [1.22; 2.47]; Fig. 2a), cardiac death (RR 2.44 [1.73; 3.44]; Fig. 2b) and bleeding (RR 1.64 [1.35; 1.98]; Fig. 2f) as well as one-year all-cause death (RR 2.62 [1.2; 5.73]; Fig. 2c) and cardiac death (RR 1.89 [1.25; 2.86]; Fig. 2d) in the cancer group compared to the non-cancer group in ACS studies. Long-term all-cause death did not significantly differ (Fig. 2e) between groups. The consistency was low for the in hospital and one-year all-cause death outcomes (I^2^ = 87 and 98% respectively). When the matched group of the study by Wang et al. [13] was used, comparisons showed important heterogeneity for in-hospital and long-term all-cause and cardiac death (Additional file 1: Figure S1).

**Fig. 3:**
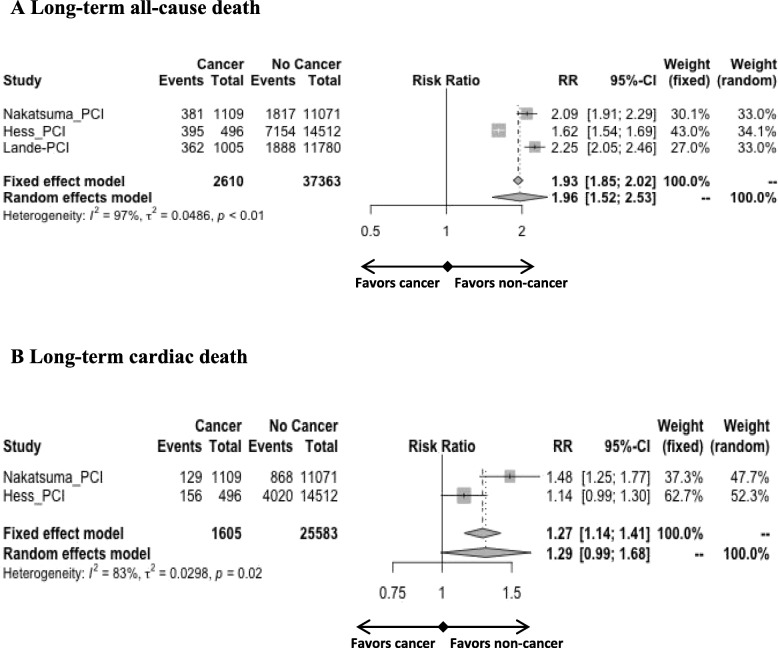
Forest plots of PCI studies comparing the impact of cancer versus no cancer on long-term all-cause death (**A**) and long-term cardiac death (**B**)

In the PCI studies, the meta-analysis showed only increased rates of long-term all-cause death (RR 1.96 [1.52; 2.53]; Fig. 3a) in the cancer group but with very low consistency (I^2^ = 97%). Long-term cardiac death did not significantly differ between groups (Fig. 3b).

Funnel plots showed publication bias for in-hospital and one-year all-cause death but not for cardiac death in all analyses and in-hospital bleeding (Additional file 1: Figure S2). The studies were judged to be at intermediate or low risk of bias using the adapted Newcastle-Ottawa Scale (Additional file 2).

## Discussion

Our analysis shows that overall 8.1% of the patients admitted for an ACS have a history of cancer. Such patients are at higher risk of in-hospital and one-year, all-cause and cardiac mortality as well as in-hospital bleeding compared to those without cancer. However the results should be tempered because of high heterogeneity and publication bias with the exception of cardiac death, which was consistently increased in cancer patients. Among PCI studies, cancer patients were at higher risk of all-cause but not cardiac long-term mortality.

Cancer patients represent a growing and high-risk population in the setting of ACS. Our meta-analysis confirmed the worse, in-hospital and one-year, prognosis of cancer patients. Even if the magnitude of the relative risk of early and late all-cause mortality should be tempered by the heterogeneity among studies, all included studies consistently showed a worse prognosis in such patients. The heterogeneity among studies may be explained by the differences in sample size and statistical methods. Additionally, the small increase of all-cause death in cancer patients observed in the largest study by Gong et al. [8] -that differs from other results- is explained by their selection of cancer survivors only (without cancer treatment nor diagnosis within the last year). Patients with cancer are older, more often women and have more comorbidities including diabetes mellitus, chronic kidney disease, history of heart failure and stroke [8, 9, 11, 12], compared to non-cancer patients. These conditions are associated with poor prognosis in ACS patients [17–20]. Increased risk of mortality may also be explained by the prothrombotic state associated with cancer, due to reduced fibrinolysis and production of procoagulants -such as tissue factor- and inflammatory cytokines by the tumor [21] as well as tumor cell-induced platelet aggregation [22]. Moreover malignancy is associated with the risk of stent thrombosis [23]. Finally, patients with cancer are less likely to receive optimal guideline recommended medications [9, 11, 24].

The use of early invasive strategy, and PCI if needed, is associated with improved outcome after ACS [25, 26]. A less frequent use of PCI or drug eluting stents in patients with a history of cancer admitted for ACS has been reported [9, 11, 12] but current data remain conflicting [8]. A recent study reported that optimal medical therapy was prescribed in only one third of cancer patients at discharge [27]. The higher comorbidities associated with cancer such as renal impairment, asthenia and anemia [8, 9, 11, 16] may contribute to the suboptimal use of invasive strategy and evidence-based recommended medication. Moreover, the use of potent antithrombotic treatments may be limited in such patients because of the higher early bleeding risk found in our study. Explanations for higher bleeding risk may include the tumour-bleeding risk, the more frequent need for surgery, the drug-induced bone marrow toxicity and malnutrition [28] as well as comorbidities especially older age and renal impairment. Extensive data have shown that bleeding in ACS is associated with high risk of mortality [29, 30].

Considering the growing number of cancer patients admitted for ACS and their higher risk of death and bleeding, optimal management of these patients is crucial in clinical practice. The main cause of in-hospital mortality after ACS remains cardiac death [12, 13]. A reported, increased use of invasive coronary strategies and pharmacotherapies in such patients has been associated with a decline of mortality over the same time period [8]. A tailored approach appears important to reduce both the risk of cardiac death and bleeding during the acute phase. A multidisciplinary approach with a cardio-oncologist may be helpful for the early and long term management of such complex patient population [31]. The higher rates of long term all-cause but not cardiac death in cancer patients admitted for PCI highlights the fact that non-cardiovascular comorbidities may be of greater prognostic importance over the years after an ACS [32] as cancer patients will mostly die of cancer at long-term [13].

### Limitations

Our meta-analysis was not performed on individual patient data. We pooled together studies with a large degree of clinical heterogeneity which is mirrored by the statistical heterogeneity across some outcomes. Cancer patients are excluded from most trials and limited data are available from observational studies. This point is a limit but also a justification for our meta-analysis. Cancer drugs may have influenced the hemorrhagic risk because of their potential different interactions with antiplatelet agents but these data were not available for more detailed analysis. Finally data on the cancer type or the time interval between cancer diagnosis and ACS which may highly impact the prognosis [12] were not available for further analysis.

## Conclusion

Cancer patients represent a growing and high-risk patient population admitted for ACS. Our study showed that this population is at higher risk of in-hospital and one-year, all-cause and cardiac mortality as well as higher in-hospital bleeding risk compared to non-cancer patients. Even if the magnitude of the risk of all-cause mortality should be tempered by the heterogeneity among studies, all studies show higher risks of mortality in such patients. The consistent results with respect to the risk of cardiac mortality, especially the twice higher risk of in-hospital cardiac death, support optimal management of these patients with a tailored pharmaceutical and invasive strategy in the acute phase.

## Supplementary information

**Additional file 1: Figure S1.** Forest plots of acute coronary syndrome (ACS) studies comparing the impact of cancer versus absence of cancer on in hospital all-cause death (A), in-hospital cardiac death (B), long-term all-cause death (C) and long-term cardiac death (CD) including the matched comparison for the study of Wang et al. **Figure S2.** Funnel plots in the acute coronary syndrome studies (panel A = In-hospital all-cause death; panel B = In-hospital cardiac death; panel C = One-year all-cause death; panel D = One-year cardiac death; panel E = In-hospital bleeding). Egger’s test was not applicable for one-year cardiac death and in-hospital bleeding as there were only 2 studies.

**Additional file 2: Table S1.** Newcastle-Ottawa Scale for risk of bias assessment of studies included in the meta-Analysis.

## Data Availability

All data analyzed during this study are included in this published article and its supplementary information files.

## Abbreviations

ACS: Acute coronary syndrome
CI: Confidence intervals
PCI: Percutaneous coronary intervention
PRISMA: Preferred reporting items for systematic reviews and meta-analysis
RR: Relative risks
