# Supplementary material for: Systematic review and meta-analysis of the prognostic impact of cancer among patients with acute coronary syndrome and/or percutaneous coronary intervention
Source: BMC Cardiovasc Disord. 2020 Jan 30;20:38. doi: 10.1186/s12872-020-01352-0 (PMC6993442; doi:10.1186/s12872-020-01352-0)
Supplement: Supplementary file 1 — Additional file 1: Figure S1. Forest plots of acute coronary syndrome (ACS) studies comparing the impact of cancer versus absence of cancer on in hospital all-cause death (A), in-hospital cardiac death (B), long-term all-cause death (C) and long-term cardiac death (CD) including the matched comparison for the study of Wang et al. Figure S2. Funnel plots in the acute coronary syndrome studies (panel A = In-hospital all-cause death; panel B = In-hospital cardiac death; panel C = One-year all-cause death; panel D = One-year cardiac death; panel E = In-hospital bleeding). Egger’s test was not applicable for one-year cardiac death and in-hospital bleeding as there were only 2 studies. [file 12872_2020_1352_MOESM1_ESM.docx]

**Supplementary File 1**

**Figure S1:** Forest plots of acute coronary syndrome (ACS) studies comparing the impact of cancer versus absence of cancer on in hospital all-cause death (A), in-hospital cardiac death (B), long-term all-cause death (C) and long-term cardiac death (CD) including the matched comparison for the study of Wang et al.

**S1-A In-hospital all-cause death**

**
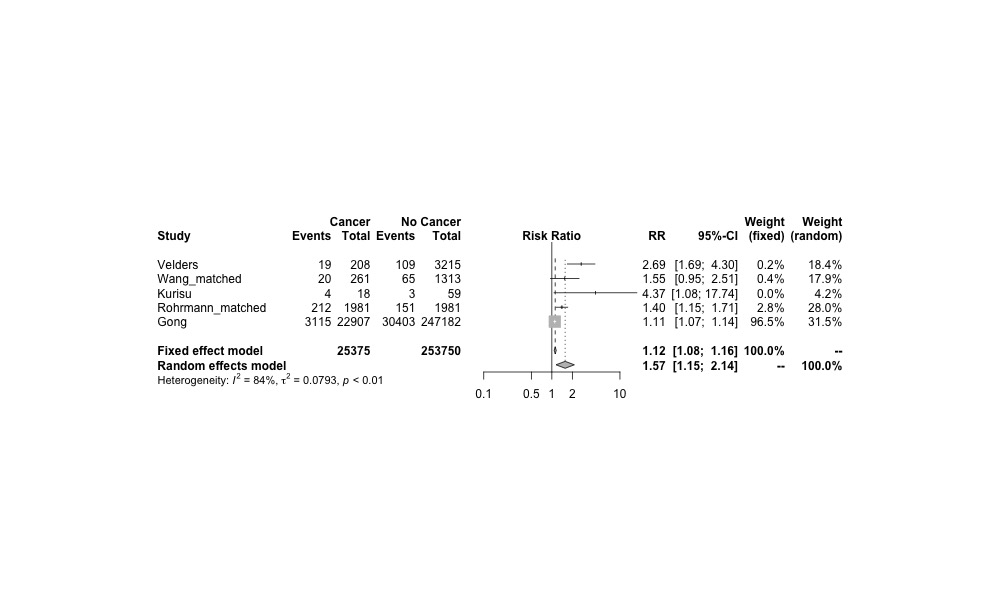
**

**Favors non-cancer**

**Favors cancer**

**S1-B In-hospital cardiac death**

**
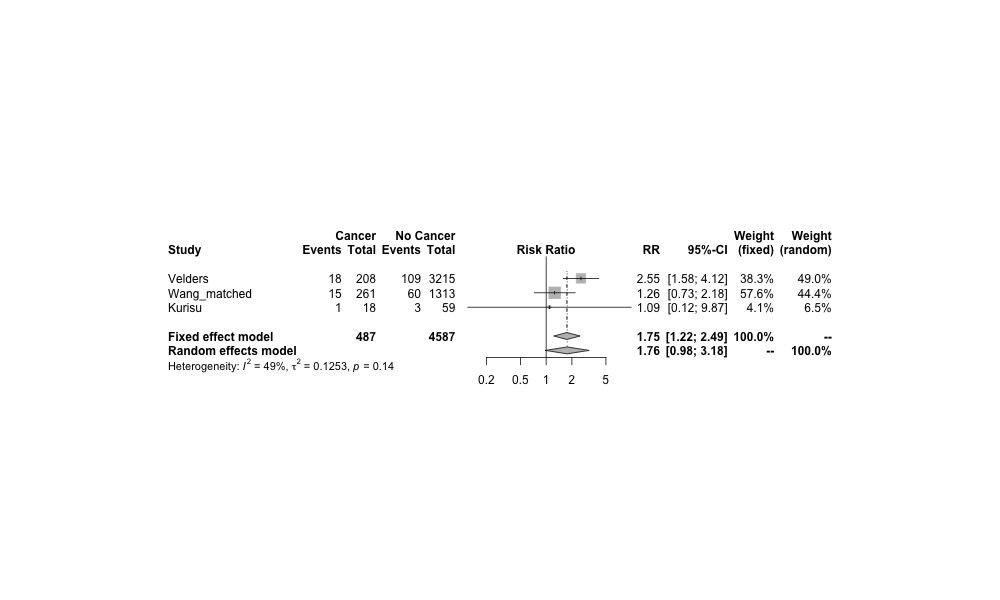
**

**Favors non-cancer**

**Favors cancer**

**S1-C Long-term all-cause death**

**
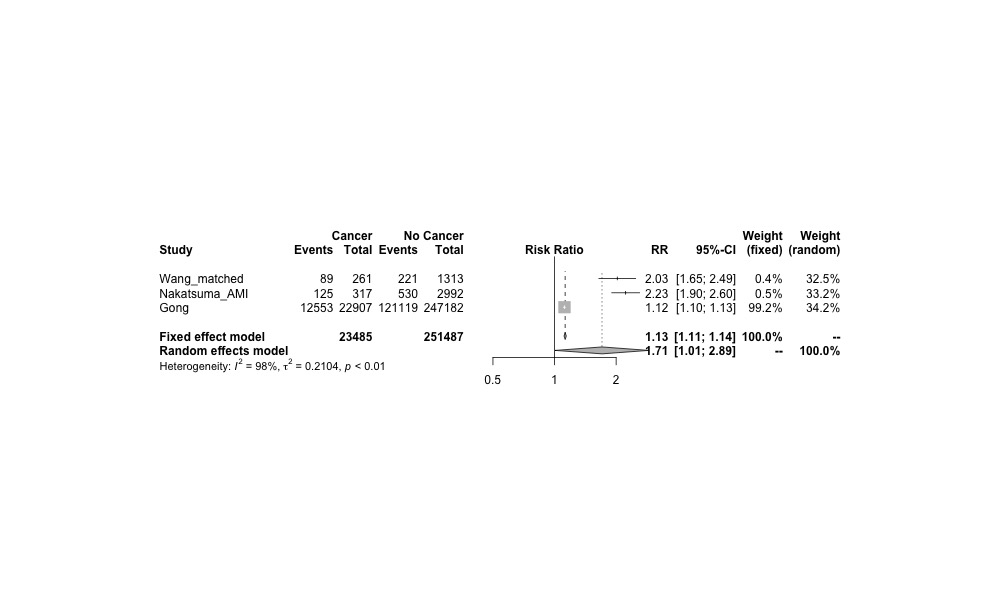
**

**Favors non-cancer**

**Favors cancer**

**S1-D Long-term cardiac death**


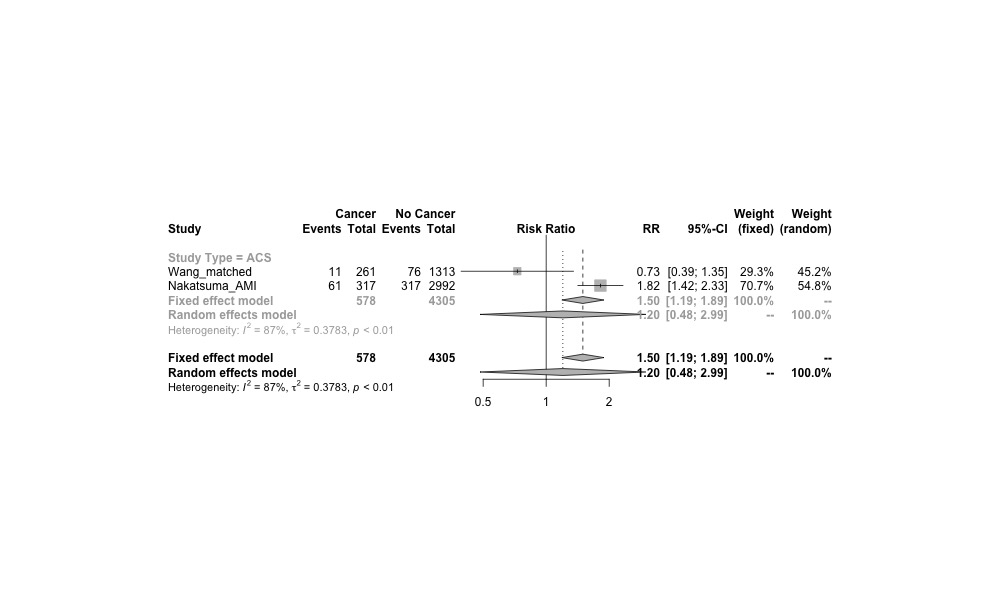

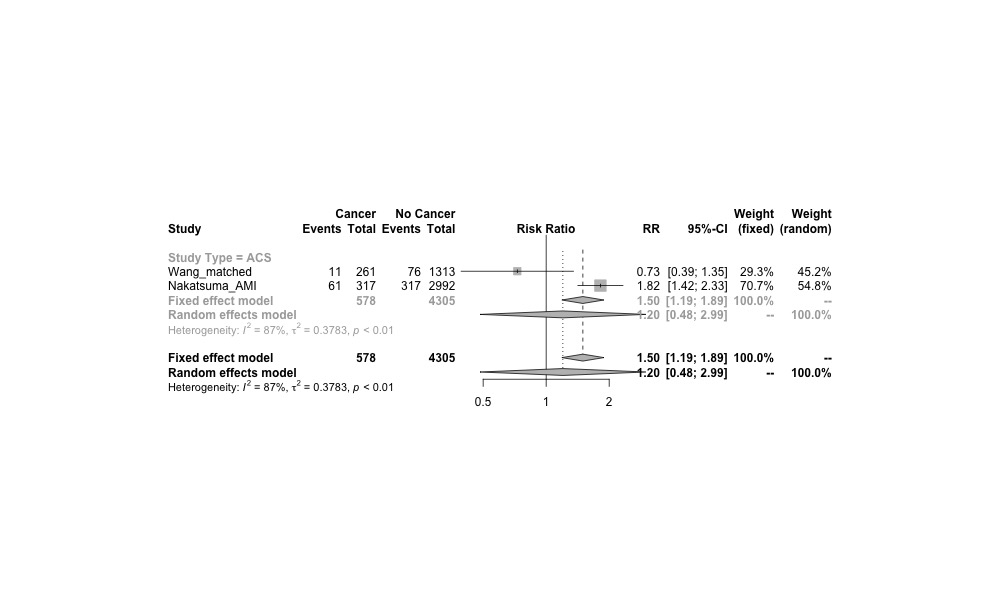

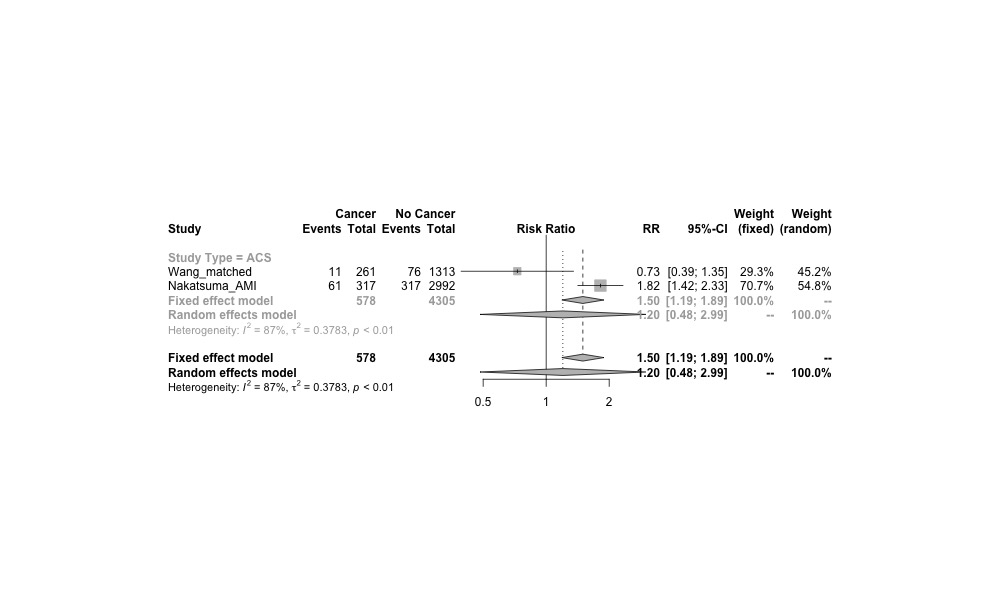


**Favors non-cancer**

**Favors cancer**

**Figure S2:** Funnel plots in the acute coronary syndrome studies (panel A = In-hospital all-cause death; panel B = In-hospital cardiac death; panel C = One-year all-cause death; panel D = One-year cardiac death; panel E = In-hospital bleeding). Egger’s test was not applicable for one-year cardiac death and in-hospital bleeding as there were only 2 studies.

**S2-A In-hospital all-cause death (p**=**0.007 suggesting possible publication bias)**

**
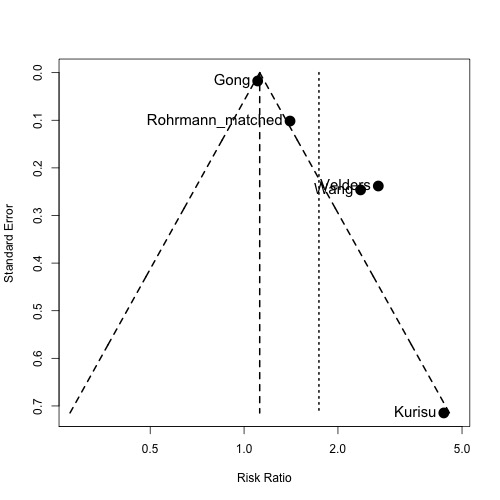
**

**S2-B In-hospital cardiac death (p=0.09)**

**
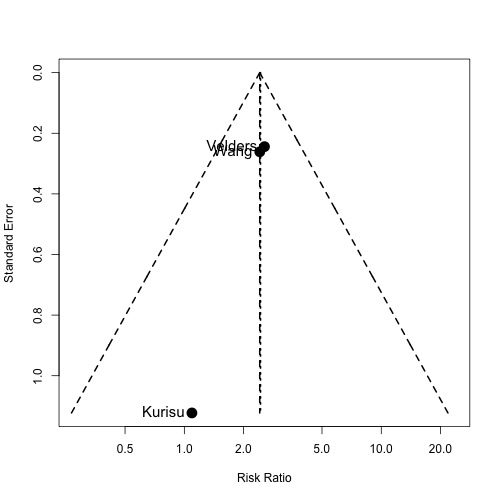
**

**S2-C One-year all-cause death (p=0.1)**

**
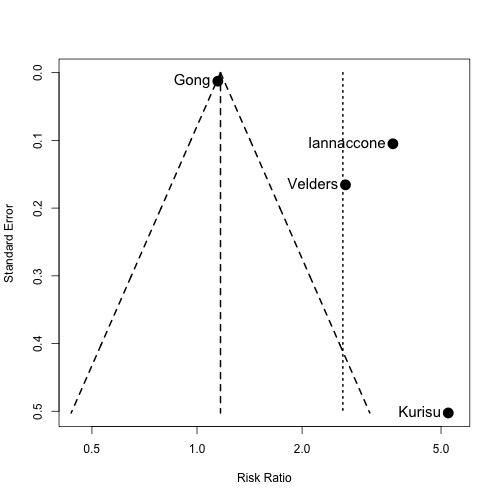
**

**S2-D One-year cardiac death**

**
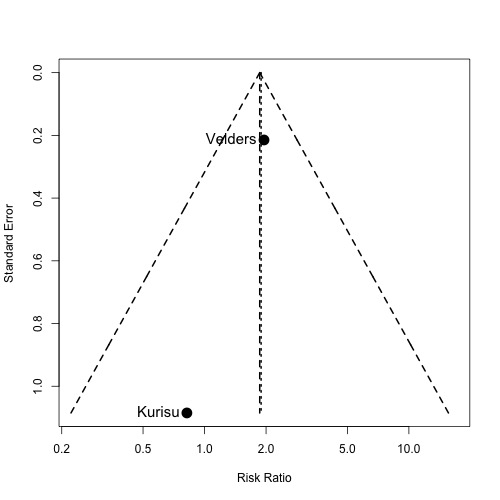
**

**S2-E In-hospital bleeding**

**
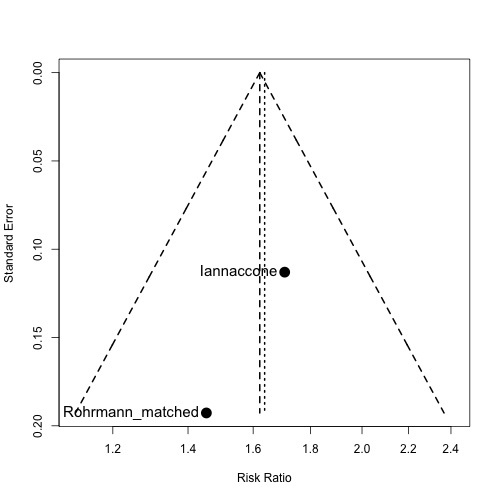
**
