# Supplementary material for: Systematic review and meta-analysis of the prognostic impact of cancer among patients with acute coronary syndrome and/or percutaneous coronary intervention
Source: BMC Cardiovasc Disord. 2020 Jan 30;20:38. doi: 10.1186/s12872-020-01352-0 (PMC6993442; doi:10.1186/s12872-020-01352-0)
Supplement: Supplementary file 2 — Additional file 2: Table S1. Newcastle-Ottawa Scale for risk of bias assessment of studies included in the meta-Analysis. [file 12872_2020_1352_MOESM2_ESM.docx]

**Supplementary File 2**

**NEWCASTLE - OTTAWA QUALITY ASSESSMENT SCALE**

**Adapted to our analysis**

Note: A study can be awarded a maximum of one point for each numbered item within the Selection and Exposure categories. A maximum of two points can be given for Comparability.

**Selection**

1) Is the case definition (cancer) adequate?

a) yes, with independent validation (eg. self-reported doctor’s diagnosis, reference to primary record source) => 1 point

b) yes, based on self-reports

c) no description

2) Representativeness of the cases

a) consecutive or obviously representative series of cases => 1 point

b) potential for selection biases or not stated

3) Selection of Controls

a) same community as cases (admitted for an acute coronary syndrome and /or PCI) => 1 point

b) no description

4) Definition of Controls

a) no history of disease => 1 point

b) no description of source

**Comparability**

1) Comparability of cases and controls on the basis of the design or analysis

a) study controls for age => 1 point

b) study controls for gender and smoking => 1 point

**Exposure**

1) Ascertainment of exposure

a) all-cause or cardiac death (based on hospital or municipality records) => 1 point

b) no validation is mentioned

c) no description

2) Same method of ascertainment for cases and controls

a) yes => 1 point

b) no

**Table S1:** Newcastle-Ottawa Scale for risk of bias assessment of studies included in the meta-Analysis

| **Studies** | **Selection** | | | | **Comparability** | | **Exposure** | | **Total (maximum 8)** |
| --- | --- | --- | --- | --- | --- | --- | --- | --- | --- |
|  | Case definition adequate | Representativeness of the cases | Selection of controls | Definition of controls | Comparability : age | Comparability : gender | Ascertainment of exposure | Comparable ascertainment method |  |
| Velders, 2013 |  | 1 | 1 | 1 | 1 | 1 | 1 | 1 | 7 |
| Rohrmann, 2018 | 1 | 1 | 1 | 1 | 1 | 1 | 1 | 1 | 8 |
| Gong, 2018 | 1 |  | 1 | 1 | 1 | 1 | 1 | 1 | 7 |
| Iannaccone, 2018 | 1 |  | 1 | 1 |  |  | 1 | 1 | 5 |
| Kurisu, 2013 | 1 |  | 1 | 1 |  |  | 1 | 1 | 5 |
| Wang, 2016 | 1 | 1 | 1 | 1 | 1 | 1 | 1 | 1 | 8 |
| Hess, 2015 | 1 | 1 | 1 | 1 | 1 | 1 | 1 | 1 | 8 |
| Landes, 2017 | 1 | 1 | 1 | 1 | 1 | 1 | 1 | 1 | 8 |
| Nakastuma, 2018 | 1 | 1 | 1 | 1 | 1 | 1 | 1 | 1 | 8 |
